# The Influence of Bovine Milk High or Low in Isoflavones on Hepatic Gene Expression in Mice

**DOI:** 10.1155/2010/423179

**Published:** 2010-06-28

**Authors:** Mette T. Skaanild, Tina S. Nielsen

**Affiliations:** ^1^Department of Disease Biology, Faculty of Life Sciences, University of Copenhagen, 1870 Frederiksberg C, Denmark; ^2^Department of Animal Health and Bioscience, Faculty of Agricultural Sciences, Aarhus University, 8830 Tjele, Denmark

## Abstract

Isoflavones have generated much attention due to their potential positive effects in various diseases. Phytoestrogens especially equol can be found in bovine milk, as feed ration for dairy cows is comprised of plants containing phytoestrogens. The aim of this study was to analyze the changes in hepatic gene expression after dietary intake of milk high and low in isoflavones. In addition to pelleted feed female NMRI mice were offered water, water added either 17*β*-estradiol, equol, Tween 80, and milk high and low in isoflavone content for a week. Gene expression was analyzed using an array qPCR kit. It was revealed that Tween 80 and 17*β*-estradiol upregulated both phase I and phase II genes to the same extent whereas equol alone, high and low isoflavone milk did not alter the expression of phase I genes but decreased the expression of phase II genes. This study shows that dietary isoflavones can regulate the transcription of especially phase II liver enzymes which potentially could give rise to an increase in reactive oxygen metabolites that may contribute to the development of cancer.

## 1. Introduction

In recent years isoflavones (a group of phytoestrogens) have generated much attention both in science and in public due to their potential health beneficial effects in relation to diseases such as atherosclerosis, breast, endometrial, and prostate cancer, but also osteoporosis reviewed by Duncan et al., 2003 [1]. 

Especially leguminous plants, such as clover and soya bean, have high natural contents of phytoestrogens, the most predominant being daidzein, genistein, formononetin, and biochanin. These phytoestrogens can be found in bovine milk [2], as a large proportion of the feed ration for dairy cows is comprised of leguminous plants. Apart from these plant isoflavones, equol may also be present in bovine milk. Equol is produced from daidzein in the rumen of cows and the human intestine by the micro flora [3, 4]. Equol is of special interest since it may directly exert cancer preventive effects [5]. However, only about one third of people in the Western populations can produce equol, likely due to the large interindividual variability in the intestinal flora. 

As isoflavones can change the phase 1 and phase 2 metabolisms [6], they have the potential to change the metabolism of endogenous compounds such as hormones and xenobiotic compounds including drugs. This may cause a change in homeostasis and/or change the oxidative tress of liver which may give rise to cancer. Most of these studies, however, have been made *in vitro*. A recent in vivo study revealed that some dietary isoflavones could change the oxidative metabolism of 17*β*-estradiol (E2) measured using microsomes from the levers, but the activity of CYP1A, CYP2B, and CYP3A did not change [7] indicating that the changed metabolism of E2 could be caused by an altered activity of flavin-containing monooxygenases (FMOs).

The objective of this study was to investigate if bovine milk high or low in isoflavones can alter the liver phase 1 and phase II metabolism. The effect of purified equol and E2 was also studied to test if the effects caused by isoflavones were an estrogen-like modification. As Tween 80 (polyxyoethylene sorbitan monooleate) was used as a solvent for E2 it was also tested. Both E2 and Tween 80 were administered via drinking water. The effect was measured as changes in transcription of an array of liver enzymes coding for both phase I and phase II enzymes and transporters.

## 2. Material and Methods

### 2.1. Milk and Chemicals

The high and low isoflavone milk (HIM and LIM) tested in the present experiment were obtained from a grazing study described in detail by Eriksen et al. [8]. Bulk milk was obtained at the end of two 3-week periods in May to June 2006 from cows grazing alfalfa (*n* = 12) (low isoflavon milk, LIM) and red clover (*n* = 12) (high isoflavone milk, HIM) was pooled. The fat percentage of the milk was determined according to the method described earlier [9], and milk was standardized to approximately 1.5% fat to simulate low-fat milk. The crude protein content was determined as Dumas *N* × 6.25 (3.4 and 3.3% for HIM and LIM, resp.). Milk was pasteurized in water bath (15 min, 65°C) and stored at −20°C until further use. 

 The analysis of the milk for isoflavones was done as described by Nielsen et al. [10]. Milk samples were equilibrated to room temperature before the addition of 25 *μ*L prunetin (120 ng/mL) as internal standard and 100 *μ*L *β*-glucuronidase in order to extract aglycones of phytoestrogens. The mixture was incubated at 37°C for 1 hour and shaken vigorously every 15 minutes. After centrifugation (10 min at 4000 rpm), both the creamy layer and the precipitate were discarded, while the liquid phase was recovered and submitted to solvent extraction with hexane (3 × 3 mL) followed by ethyl acetate (3 × 3 mL). The ethyl acetate extract was evaporated to dryness under a flow of nitrogen. The residue was then resuspended in 500 *μ*L acetonitrile and a LC-MS analysis was performed [10]. 

Cyclodextrin-encapsulated water soluble-17*β*-estradiol (E2) was purchased from Sigma-Aldrich A/S (Brøndby, Denmark). Racemic equol (purity ~99%) was purchased from Gentaur Molecular Products BVBA (Brussels, Belgium). 1% Tween 80 was added to dissolve equol in water and also tested.

### 2.2. Animals and Treatment

NMRI (BomTac:NMRI) outbred albino dams with litters standardized at postnatal day one to contain 11 female pups born on the same day were purchased from Taconic (Lille Skensved, Denmark). The pups were seven to eight days old on arrival. Animals were delivered pooled (four dams with 44 pups), and a dam was randomly assigned 10-11 pups and housed individually under controlled lighting (12 h light/dark cycles) and temperature (21-22°C) conditions. The dams were given fresh water and fed the RM1 diet (Special Diets Services, Witham, UK) ad libitum until the pups were weaned at day 15. The RM1 diet was chosen because of the low content of phytoestrogens compared with other standard rodent diets [11]. At weaning, pups were weighed and randomly placed in cages containing 6–10 pups per cage and randomly assigned to test groups. All mice had free access to the RM1 diet and milk or water. Mice receiving the milk treatments had fresh milk daily. The mice were euthanized by dislocation of the neck day 22 and a piece of the liver was taken out and transferred to RNA later for the gene expression analysis (Quiagen, Ballerup, Denmark). The experiment and all procedures related to the handling of animals were approved by the Danish Experimental Animal Inspectorate. 

In experiment 1, mice were allocated to one of the following treatments: Water alone (Control) (*n* = 9); low isoflavone milk (LIM, *n* = 15); high isoflavone (HIM, *n* = 15); 33 or 100 ng E2/mL drinking water (*n* = 8). In experiment 2 the treatments were as follows: Water alone (Control, *n* = 10); 50 *μ*g/mL equol added to the drinking water containing 1% Tween 80 (polyxyoethylene sorbitan monooleate) (*n* = 10) to dissolve the equol. To test the possibility that the inclusion of 1% Tween 80 in the drinking water could affect the water intake or gene expression, an additional group of 10 mice were given drinking water containing only 1% Tween 80.

### 2.3. Liver Gene Expression Analysis

Total RNA was isolated from 8 animals from each group both from experiments 1 and 2. The RNA was isolated using the Nucleospin Kit (Macherey Nagel, Duren, Germany) following the manufacturer's manual. The reverse transcription and PCR were set up using RT^2^ First strand Kit and RT^2^ SYBR Green qPCR Master Mix from SABiosciences (Tebu-bio Aps. Roskilde, Denmark). The reactions were set up according to the Kit manuals.

The gene expression analysis was carried out using Mouse Drug Metabolism array assay from SABiosciences. Two array plates were set up for each group of animals (4 animals/plate). For each array, 4 ug total RNA was used, 1 ug of total RNA from each animal. In the mouse drug metabolism array, gene expression of 84 genes involved mainly in phase I and phase II metabolism can be analyzed (Table 1). The array also includes negative and positive control and the following 5 housekeeping genes: *β* glucuronidase, hypoxyanthine guanine phosphoribosyl transferase 1, heat shock protein 90 *α*, glyceraldehyde-3-phosphate dehydrogenase, and *β* actin in order to calculate expression changes. The average Cp values for all 5 housekeeping genes were used to estimate the expression changes. Software developed by SABiosciences especially for these arrays was used to calculate the changes in gene expression.

## 3. Results

### 3.1. Phytoestrogens and Endogenous Estrogens in Milk

The concentrations of isoflavones varied between the HIM and LIM. Especially for the isoflavone metabolite equol the differences were very pronounced, 1003 ng/mL and 57,4 ng/mL for HIM and LIM, respectively (Table 2). Mainly due to these differences, the total cumulative concentrations also varied significantly between HIM (1215 ng/mL) and LIM (118 ng/mL). The endogenous concentration of estrogens in milk, however, did not vary significantly (Table 2). 

From these result and based on the daily intake of milk in the two groups, (see Nielsen et al. [10] for further details) the total daily intake of phytoestrogens in mice was calculated to be 425 and 3767 ng/d for low and high isoflavones milk, respectively. The intake of estrogen was 323 and 186 pg/d for HIM and LIM, respectively. The equol-treated group was given 115000 ng/d of equol and the E2-treated group was exposed to 200 ng/d of estradiol.

### 3.2. Gene Expression Analysis

 The result for genes modulated more than 2 times compared to control (the group given water or water with 1% Tween 80) is presented in Table 3. One % Tween 80 (A) and 17*β*-estradiol (F) upregulated both phase I and phase II genes by 2.1–9.5 times and 2.2–25.4 times, respectively. Equol, compared to water, only upregulated 4 genes and downregulated 2 genes, but when equol was compared to 1% Tween 80, 19 genes were regulated, 17 downregulated, and 2 upregulated. HIM (D) and LIM (E) caused a decrease in expression of genes catalyzing the phase II metabolism by 2. 1–5 times. An increased expression was obtained for one gene, a transport protein (metallothionein 2) in the LIM group, but not in the HIM group**.** The average Cp for the 5 housekeeping genes in each group varied from 20.3 to 20.9.

## 4. Discussion

The potential positive effects of phytoestrogens have generated much attention by both the public and scientists, whereas other effects, that is, changes in liver metabolism have not attracted the same attention. This study is the first to look into how cow's milk differing in content of especially isoflavones affects the expression of an array of genes in the liver. The content of phytoestrogens in milk can vary as seen for HIM and LIM depending on the composition of the diet that cows are fed, and organic milk in general contains higher concentrations of isoflavones especially equol [2, 12, 13]. The amount of equol found in this studied HIM is the highest so far reported, most likely due to the large proportion of red clover in the fields where cows are grazing [10], as red clover is known to contain high amounts of the equol precursors formononetin and daidzein [13, 14].

Both E2 and 1% Tween 80 affect the liver gene expression by increasing the expression of the same phase I and phase II genes. This indicates that Tween 80 affects the liver metabolism the same way and to the same extent as 100 ng E2/mL drinking water. Equol, when compared to Tween 80 on the other hand, seems to exert an opposite effect as this isoflavone downregulates many of the same genes as E2 upregulates. Most of the genes that are modulated by equol are also altered by HIM and LIM indicating that the concentration of equol in both types of cow's milk might be high enough to exert an effect. No altered expressions were found for the phase I enzymes which are in concordance with earlier published results where the enzymatic activities of the P450 isoenzymes were found to be unchanged [7]. This indicates that the enzyme activity of these enzymes is potentially only regulated (inhibited) at protein level. Unfortunately, the decrease in expression of the following phase II genes Chst1 (a sulfotransferase), Ggt (a glutamyltransferase), Gstp1 (a gluthathione S- transferase), and Nat1(a acetyltransferase) may have a negative effect. A decrease in enzymes catalysing the conjugation reactions may cause an increase in the concentration of bioactivated compounds in the liver. As these compounds can form adduct to both proteins and DNA, they may be carcinogenic. The antioxidant level in the cell may also be altered by both milk types caused by a decrease in the levels of peroxidases and decarboxylases. The oxidative stress, that this may induce, can cause cell damage as the reactive metabolites can start a destruction of the cell membrane.

 Hydroxysteroid (17-beta) dehydrogenase 1 and 3 (Hsd17b1 and Hsd17b3) catalyses the conversion of estrone to the more reactive estradiol and androstenodione to androgen testosterone, respectively [15, 16], and the concentration of this enzymes is very high in the liver. Inhibitors of Hsd17b1 are currently being developed for use in therapy of hormone-dependent breast cancers as this will give a lower concentration of the active estradiol [17]. The reduction in protein level of this enzyme caused by the milk may explain the positive effect of phytoestrogens on breast cancer found in some studies [1]. Mutations in the Hsd17b3 genes occur in humans and can cause male pseudohermaphroditism or feminization because of decreased function [18]. A decrease in the gene expression of this gene is seen after exposure to both types of milk, and it may therefore be able to induce feminization. Also development of cancers of colon, breast, uterus, endometrium, and prostate has been hypothesized to be promoted by loss of activity of Hsd17B1 and Hsd17b3 as reviewed by Vihko et al. [19].

Equol and HIM also induced a decrease in mRNA coding for amiloride-binding protein (amine oxidase, Abp1). This enzyme catalyzes the oxidative deamination compounds such as histamine [20] and may therefore affect clinical diseases related to histidin, that is, asthma and allergic rhinitis [21]. High plasma levels of this enzyme have been observed in human cancer but as the concentration of this enzyme decreases, no carcinogenic effect with regard to this system is expected.

 From this study it can be concluded, that both HIM and LIM induce changes in hepatic gene expression likely due to the presence of isoflavones since the genes affected are also affected by equol. The decrease in phase II enzymes could potentially give rise to epigenetic actions contributing to carcinogenesis by increasing the level of reactive oxygen metabolites. The use of Tween 80 in future in *vivo* studies should be considered because of its effect on hepatic metabolism.

## Figures and Tables

**Table 1:** A list of functional gene groupings included in the array assay.

*Drug Transporters:*
Metallothioneins: Mt2, Mt3.
P-Glycoprotein Family: Abcb1a, Abcb1b, Abcb4, Abcc1, Gpi1.

*Phase I Metabolising Enzymes:*
P450 Family: Cyp11b2, Cyp17a1, Cyp19a1, Cyp1a1, Cyp1a2, Cyp27b1, Cyp2b10, Cyp2C29, Cyp2e1, Cyp4b1.

*Phase II Metabolising Enzymes:*
Carboxylesterases: Ces1, Ces2.
Decarboxylases: Gad1, Gad2.
Dehydrogenases: Adh1, Adh4, Adh5, Alad, Aldh1a1, Hsd17b1, Hsd17b2, Hsd17b3.
Glutathione Peroxidases: Gpx1, Gpx2, Gpx3, Gpx5, Gsta1, Gsta3, Gsta4, Gstm1, Gstm2, Gstm3, Gstm4, Gstm5, Gstp1, Gstt1, Gstz1, Lpo,
Mpo.
Hydrolases: Ephx1, Faah, Fbp1.
Kinases: Hk2, Pklr, Pkm2.
Lipoxygenases: Alox12, Alox15, Alox5, Apoe.
Oxidoreductases: Blvra, Blvrb, Cyb5r3 (Dia1), Gpx1, Gpx2, Gsr, Mthfr, Nos3, Nqo1, Srd5a1, Srd5a2.
Paraoxonases: Pon1, Pon2, Pon3.
Sulfotransferases: Chst1, Gsta3, Gstm2, Gstm3, Gstm5, Gstp1, Gstt1, Mgst1, Mgst2, Mgst3.
Transferases: Nat1, Nat2, Comt, Ggt1.

*Other Genes Related to Drug Metabolism:* Abp1, Ahr, Arnt, Asna1, Gckr, Marcks, Smarcal1, Snn.

**Table 2:** Total concentrations (free and conjugated) of the measured phytoestrogens (ng/ml) and endogenous estrogens E1 and E2 (pg/mL) in high (HIM) and low (LIM) isoflavone milk.

	LIM	HIM
*Isoflavones*		
Formononetin*	10.7 ± 0.2	20.4 ± 0.9
Biochanin A*	1.3 ± 0.07	0
Glycitein*	5.9 ± 0.1	10.2 ± 1.0
Daidzein*	0.3 ± 0.02	5.7 ± 0.05
*Isoflavone metabolite*		
Equol*	57.4 ± 0.9	1003 ± 37
*Flavone*		
Chrysin*	0	0.4 ± 0.02
*Flavanone*		
Naringenin*	13.4 ± 0.1	16.5 ± 0.7
*Lignans*		
Matairesinol*	0	48.5 ± 5.5
Enterodiol*	4.0 ± 0.04	2.6 ± 0.2
Enterolactone*	24.6 ± 0.4	11.0 ± 1.3
*Coumestans*		
Coumestrol	0	0
Total*	118	1215

Estrone (E1)	566 ± 17	503 ± 17
17*β*-estradiol (E2)	64.6 ± 5	60.0 ± 5

*Statistically significant difference between LIM and HIM.

**Table 3:** A list of genes regulated more than 2 times up (bold) or down (italic). Regulation caused by: A Tween 1%, B equol, D HIM, E LIM, and F 17-*β* estradiol compared to control (mice feed water) and C equol compared to Tween.

A	B	C	D	E	F	Symbol	Description	Ref. seq.
**2.4**					**2.5**	Abcb1b	ATP-binding cassette, sub-family B	NM_011075
**2.7**		*2.5*	*2.5*		**3.8**	Abp1	Amiloride binding protein 1	NM_029638
					*3.2*	Aldh1a1	Aldehyde dehydrogenase family 1,	NM_013467
		*2.1*	*2.8*			Alox15	Arachidonate 15-lipoxygenase	NM_009660
**3.3**	**2.2**				**5.7**	Alox5	Arachidonate 5-lipoxygenase	NM_009662
						Blvra	Biliverdin reductase A	NM_026678
**3.4**		*4.2*		*3.1*	**4.7**	Chst1	Carbohydrate (keratan sulfate Gal-6)	NM_023850
**7.4**	**2.6**	*2.9*			**12.2**	Cyp11b2	Cytochrome P450, family 11, subfamily b.	NM_009991
**9**		*6.2*			**12.8**	Cyp19a1	Cytochrome P450, family 19, subfamily a.	NM_007810
**2.3**		*2.4*		*2.1*	**3.2**	Cyp1a1	Cytochrome P450, family 1, subfamily a,	NM_009992
**8.7**	**2.5**	*3.5*			**12.4**	Cyp27b1	Cytochrome P450, family 27, subfamily b.	NM_010009
		*2.1*				Faah	Fatty acid amide hydrolase	NM_010173
**2.5**	*2.4*	*6.1*	*5*	*4.3*	**4.3**	Gad2	Glutamic acid decarboxylase 2	NM_008078
				*2.2*		Ggt1	Gamma-glutamyltransferase 1	NM_008116
**4.6**		*3*	*2.9*		**7.1**	Gpx2	Glutathione peroxidase 2	NM_030677
					**2.4**	Gpx5	Glutathione peroxidase 5	NM_010343
						Gstm1	Glutathione S-transferase, mu 1	NM_010358
*2.1*		**2.3**	*2.1*			Gstp1	Glutathione S-transferase, pi 1	NM_013541
**2.4**		*2*			**2.7**	Hk2	Hexokinase 2	NM_013820
**2.8**		*2.1*		*2.5*	**3.8**	Hsd17b1	Hydroxysteroid (17-beta) dehydrogenase 1	NM_010475
**2.1**			*2.11*	*2.8*	**3.4**	Hsd17b3	Hydroxysteroid (17-beta) dehydrogenase 3	NM_008291
**9.5**	**3.4**	*2.8*			**15.4**	Lpo	Lactoperoxidase	NM_080420
		*2.1*	*2.2*			Mpo	Myeloperoxidase	NM_010824
**2.1**	*2.3*	*4.8*		**6.7**	**2.8**	Mt2	Metallothionein 2	NM_008630
**2.4**					**3.3**	Mt3	Metallothionein 3	NM_013603
**6.8**		*5.3*	*2.2*	*4.6*	**9.3**	Nat1	N-acetyltransferase 1 (arylamine N-acetyltransf.)	NM_008673
**2.1**		*2.1*			**2.2**	Nos3	Nitric oxide synthase 3, endothelial cell	NM_008713
**3**		*2*			**3.3**	Pkm2	Pyruvate kinase, muscle	NM_011099
**2.2**		*2.3*		*2.2*	**2.5**	Snn	Stannin	NM_009223
**2.35**					**2.3**	Srd5a2	Steroid 5 alpha-reductase 2	NM_053188
